# TiO_2_ Photocatalysis for Transfer Hydrogenation

**DOI:** 10.3390/molecules24020330

**Published:** 2019-01-17

**Authors:** Dongge Ma, Shan Zhai, Yi Wang, Anan Liu, Chuncheng Chen

**Affiliations:** 1 School of Science, Beijing Technology and Business University, Beijing 100048, China; zhaishan940@foxmail.com (S.Z.); wangyi_ii@163.com (Y.W.); 2 Basic Experimental Center for Natural Science, University of Science and Technology Beijing, Beijing 100083, China; liuanan@ustb.edu.cn; 3 Key Laboratory of Photochemistry, Beijing National Laboratory for Molecular Sciences, Institute of Chemistry, Chinese Academy of Sciences, Beijing 100190, China; ccchen@iccas.ac.cn

**Keywords:** TiO_2_, transfer hydrogenation, photocatalysis, hydrogen donor

## Abstract

Catalytic transfer hydrogenation reactions, based on hydrogen sources other than gaseous H_2_, are important processes that are preferential in both laboratories and factories. However, harsh conditions, such as high temperature, are usually required for most transition-metal catalytic and organocatalytic systems. Moreover, non-volatile hydrogen donors such as dihydropyridinedicarboxylate and formic acid are often required in these processes which increase the difficulty in separating products and lowered the whole atom economy. Recently, TiO_2_ photocatalysis provides mild and facile access for transfer hydrogenation of C=C, C=O, N=O and C-X bonds by using volatile alcohols and amines as hydrogen sources. Upon light excitation, TiO_2_ photo-induced holes have the ability to oxidatively take two hydrogen atoms off alcohols and amines under room temperature. Simultaneously, photo-induced conduction band electrons would combine with these two hydrogen atoms and smoothly hydrogenate multiple bonds and/or C-X bonds. It is heartening that practices and principles in the transfer hydrogenations of substrates containing C=C, C=O, N=O and C-X bond based on TiO_2_ photocatalysis have overcome a lot of the traditional thermocatalysis’ limitations and flaws which usually originate from high temperature operations. In this review, we will introduce the recent paragon examples of TiO_2_ photocatalytic transfer hydrogenations used in (1) C=C and C≡C (2) C=O and C=N (3) N=O substrates and in-depth discuss basic principle, status, challenges and future directions of transfer hydrogenation mediated by TiO_2_ photocatalysis.

## 1. Introduction

**Scheme 1:**
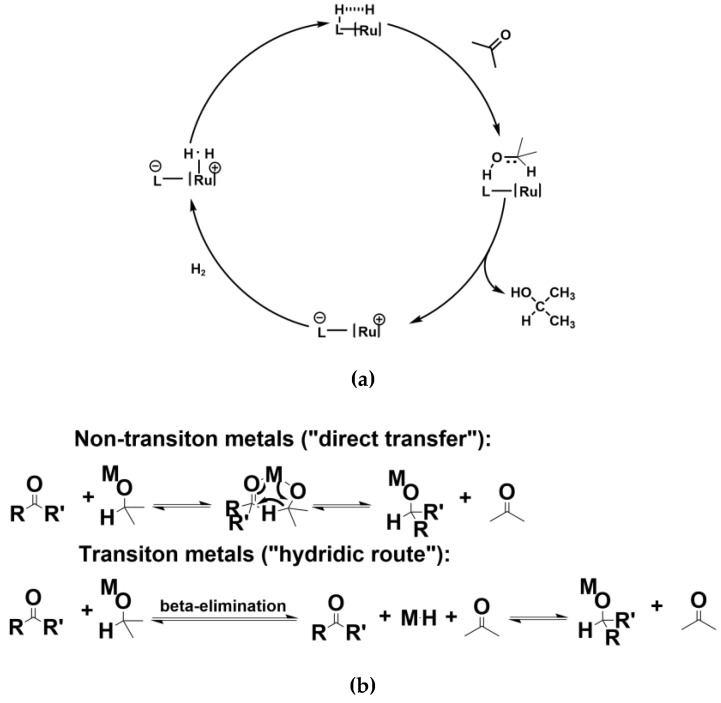
Catalytic hydrogenation (**a**) activating gaseous H_2_ as a hydrogen source (**b**) activating alcohols as a hydrogen source.

Hydrogenation of organic compounds is one of the basic transformations in organic synthesis [1,2,3]. In both laboratories and industries, hydrogenation processes are irreplaceable for producing bulk-chemicals, fine-chemicals, pharmaceuticals, agrochemicals and fragrances [4,5,6]. According to the types of hydrogen donor, hydrogenation can be roughly divided into two main categories: (i) Hydrogenation using gaseous dihydrogen [6]; (ii) transfer hydrogenation using hydrogen donors other than dihydrogen [7,8]. In consideration of the hazard and inconvenience to apply explosive gaseous dihydrogen cylinder, using safer hydrogen sources such as alcohols and amines is especially desired. Moreover, the activation of gaseous dihydrogen often requires expensive and toxic transition-metal complexes. These catalytic systems often need harsh conditions such as high reflux temperature and hydrogen pressure. Compared with hydrogenation using dihydrogen, transfer hydrogenation using alcohols and amines as hydrogen donor compounds (HDC) is safer and more convenient. Although the atom efficiency of hydrogenation using dihydrogen is higher than transfer hydrogenation, the advantage in safety and mildness made the latter a more preferential choice in laboratory and industry for the reduction of organic compounds. Recently, transfer hydrogenations have been greatly developed. Among all of the current transfer hydrogenations, catalytic transfer hydrogenations have overwhelmingly come to dominate the field in place of stoichiometric transformations. Catalytic hydrogenation processes based on both use of H_2_ and other HDC can be briefly described as Scheme 1 [9,10]. In Scheme 1a, transition-metal complexes such as the Ru complex heterolytically cleaves the dihydrogen H-H bond and the in-situ generated Ru-H complex attacks the carbonyl C=O in a nucleophilic manner yielding a Ru-alkoxide species. The following solvatolysis provides the alcohol product and regenerates the Ru complex. In Scheme 1b, both the non-transition-metal mediated direct hydrogen transfer and the transition-metal catalyzed hydridic route for transfer hydrogenation is shown. The original version of the former (the top half of Scheme 1b) was the aluminum isopropoxide mediated transfer hydrogenation named as Meerwein-Ponndorf-Verley (MPV) reduction [11,12,13]. In this case, the reaction mechanism is proposed to proceed through a six-membered transition state, without the involvement of metal hydride intermediates (the top half of Scheme 1b). The process can also be run in the opposite direction, which is the well-known Oppenauer oxidation [14]. In the transition metal case (the bottom half of Scheme 1b), it is believed that the reaction involves the formation of a metal hydride intermediate. In this case, the transition-metal complex catalyst facilitates the formation of metal-alkoxide with alcohol substrate. The alkoxide then undergoes a β-hydride elimination to give a metal-monohydride, which attacks another substrate ketone in a nucleophilic manner to realize the transfer hydrogenation [10,15]. Such a hydride intermediate has indeed been isolated in some transition-metal catalyzed transfer hydrogen reactions. Furthermore, according to the property of catalysts, catalytic transfer hydrogenations can be divided into homogeneous and heterogeneous categories. The former has garnered considerable success in the control of chemo-, regio- and even enantioselectivity [7,16]. As a significant branch of catalytic transfer hydrogenations, heterogeneous catalysis owns its distinct advantages: Easier separation and recyclization, less catalyst residue and non-decreased catalytic reactivity after multiple uses [17,18,19,20,21]. Heterogeneous catalysts play a pivotal role in the production of fine- and bulk-chemicals applying transfer hydrogenation. Specifically, heterogeneous catalytic systems have occupied a prominent status for transfer hydrogenation [22].

**Scheme 2:**
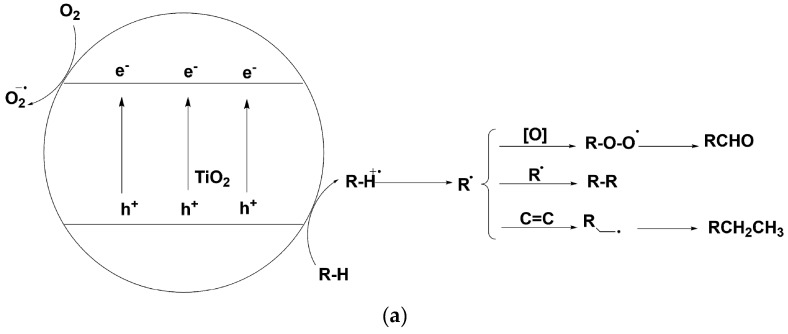

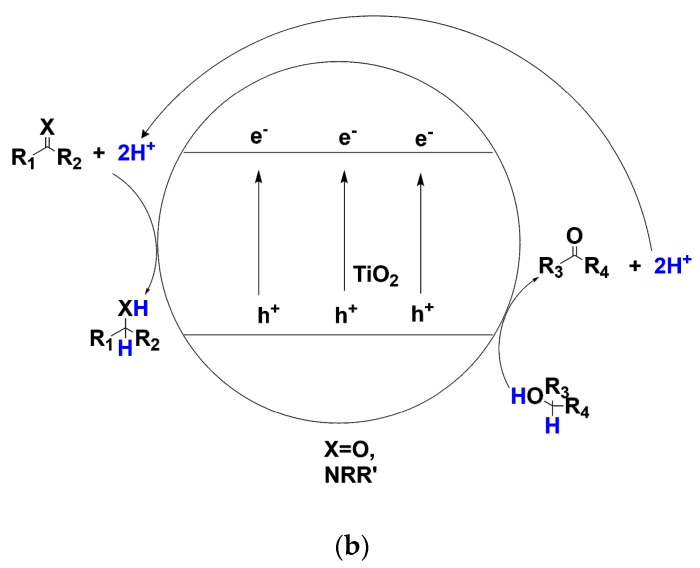
(**a**) TiO_2_ photo-induced valence-band holes and conduction-band electrons for different interfacial reactions under irradiation (**b**) TiO_2_ photocatalytic transfer hydrogenation using alcohol as hydrogen donor compound for carbonyl C=O and imine C=N reduction to alcohol and amine compounds.

As a typical heterogeneous photocatalyst, TiO_2_ nanoparticle has been thoroughly investigated and applied in a broad range of energy and environmental fields [23]. For example, TiO_2_ nanocrystal materials have been widely applied in photocatalytic water-splitting process [24], dye-sensitized-solar-cells [25], perovskite solar-cells [26] and photocatalytic detoxification of water systems and air cleaning [27,28]. Recently, the potential of TiO_2_ in organic synthetic photoredox catalysis has been discovered [29,30,31,32,33]. Upon UV, sunlight or even visible-light irradiation, the photo-induced valence-band hole and conduction-band electron on TiO_2_ surface would participate in their separate oxidative or reductive reaction with suitable electron donors or acceptors (as shown in Scheme 2a). Benefiting from this separated interfacial electron transfer, a number of TiO_2_ photocatalytic organic transformations have been unearthed. If photo-induced conduction-band electrons are consumed with the suitable electron acceptors such as dioxygen, *p*-benzoquinone, Ag^+^ cation or H^+^ upon metallic Pt loading, valence-band holes would oxidize the organic substrate furnishing the oxidative transformations. TiO_2_ photocatalysis has demonstrated its potential in a series of oxidative transformations such as alcohol oxidation to aldehydes and ketones [34,35,36,37], amine oxidative coupling to imines [38,39,40,41] and sulfide oxidation to sulfoxides [42,43]. Furthermore, TiO_2_ photocatalysis could be applied in redox-neutral C-C bond formation reactions [44,45,46]. Under appropriate light irradiation, TiO_2_ photo-induced hole which is highly oxidative possessing a redox potential as high as 2.7 V vs. normal hydrogen electrode (NHE) is generated. The hole could cleave the organic compounds’ C-X/H bonds generating radical cation species. Theses radical cations can be further trapped by unsaturated compound participating in an addition reaction to construct C-C bonds or cross-coupled with another radical species (see Scheme 2a) [44,45]. In these C-C construction transformations, both valence-band hole and conduction-band electron half-reaction are fruitful for synthesizing the final product. According to the bond being cleaved, the C-C bond formation by TiO_2_ photocatalysis are divided into the following categories: (1) Cleavage C-Si bonds [47]; (2) C-COOH bonds [48,49]. (3) C-H bond at α-position of tetrahydropyrrole [50,51]. (4) C-H bond at aldehyde α-position [52]. (5) C-H bond at both benzylic and amine α-position of *N*-arylisoquinoline [53]. (6) inert sp^2^ C-H bond of pyridine α-position [54]. Apart from oxidative and redox-neutral transformations, TiO_2_ photo-induced conduction-band electron with a redox potential of −0.5 V vs. NHE has been proved to be a mild and selective catalyst for reductive transformation [55,56,57,58,59]. In the presence of excess amount of alcohols or amines, TiO_2_ photo-induced holes would migrate to the surface and oxidize the alcohols and amines which adsorbed on TiO_2_ surface and be quenched. The depletion of valence-band holes increases the reaction rate of conduction-band electron’s reductive transformations, since this consumption of holes decreases the possibility of hole-electron recombination. In this way, TiO_2_ photo-induced conduction-band electrons would have a much longer lifetime to participate in the interfacial reduction of organic compounds.

Compared with transition-metal catalyzed transfer hydrogenation, TiO_2_ photocatalysis could achieve similarly high selectivity and yield, but usually in much milder ambient conditions and even visible-light irradiation. In recent years, some studies have uncovered that under strictly controlling anaerobic conditions, photo-induced holes on TiO_2_ nanoparticle surface in the suspending solution have the capacity to oxidatively remove hydrogens from alcohols or amines and deliver these hydrogens to the conduction band of TiO_2_ where the hydrogens combined with the conduction-band electrons and further selectively transferred to the unsaturated compounds such as carbonyls, imines multiple bonds(see Scheme 2b) [60,61,62,63]. It is heartening that practices and principles of TiO_2_ photocatalysis in these transfer hydrogenations of substrates containing C=C, C=O, N=O and C-X bond have overcome a lot of the traditional thermocatalysis’ limitations and flaws originating from high temperature operations. Being environmentally benign, photo-, acid- and basic stable, extremely convenient in separation and recyclization, TiO_2_ photocatalysis seems to be an ideal choice for transfer hydrogenations if scaling-up and enantioselectivity control issues are solved. Moreover, accompanying with the elucidation of the detailed mechanism and pathway by trapping the reaction intermediates and determining H/D kinetic isotope effects in the process of simultaneous oxidation of hole-scavenger and reduction of target organic substrate, the efficiency and the chemo-, regio- and even enantioselectivity of this methodology would be greatly improved. In this article, according to the unsaturated bond being reduced by TiO_2_ photocatalyst, this review is mainly divided into the following parts: (1) Transfer hydrogenation of C=C and C≡C (2) C=O and C=N (3) N=O. As the hydrodehalogenation by TiO_2_ photocatalysis has been recently reviewed by Zhao et al [64], this review would not cover this section.

## 2. Transfer Hydrogenation of C=C and C≡C Bonds

Catalytic reduction of C=C to C-C bonds are very important transformations in synthetic organic chemistry [65,66]. In drugs, agrochemicals and fine-chemicals structure, sp^3^C-sp^3^C moiety is ubiquitous and indispensable [67,68]. Saturated cyclic structures become more and more important in the discovery of new drug molecules [69]. With the requirement for eco-sustainable chemistry, green and mild catalytic hydrogenation process of C=C to C-C bonds is extremely desired. Heterogeneous TiO_2_ photocatalysis provides a feasible plan.

**Scheme 3:**
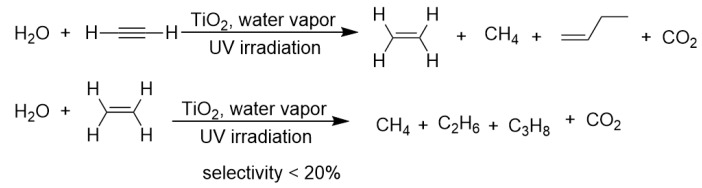
TiO_2_ photocatalyzed transfer hydrogenation of ethylene and acetylene using water vapor as a hydrogen source.

Not much later than Fujishima’s groundbreaking report on TiO_2_ photocatalysis application in water-splitting [70], Boonstra et al. discovered that TiO_2_ could act as a catalyst for the photohydrogenation of gaseous ethylene and acetylene [71]. They described that when TiO_2_ powder was degassed with ethylene or acetylene and illuminated by near-UV (ultra-violet) light, hydrogenated paraffin products were generated. The authors demonstrated that surface Ti-O-H provided reductive H-species to hydrogenate acetylene and ethylene. Kubokawa et al. discovered that after water vapor adsorption, TiO_2_ became a more effective catalyst for photohydrogenation of short-chain alkynes and alkenes. The main products were alkanes and bond fission products. Although the efficiency and selectivity were fairly low, this report spurred the further research on TiO_2_ photocatalyzed transfer hydrogenation of unsaturated compounds (as shown in Scheme 3) [72]. 

**Scheme 4:**
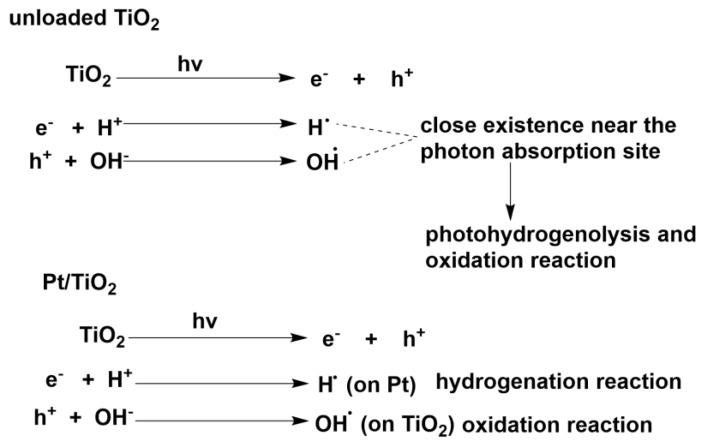
The different reaction pathways on TiO_2_ photocatalyzed hydrogenation of alkynes and alkenes on unloaded and Pt-loaded TiO_2_.

Further investigation over the reaction process by electron-paramagnetic-resonance (EPR) experiments and other mechanistic studies proved that it was H_2_O not surface Ti-OH which reduced alkynes and alkenes [73]. Furthermore, Anpo et al. discussed the influence of Pt-loading to the efficiency and product distribution of this reaction using water vapor as a hydrogen donor [74]. Using Pt-loaded TiO_2_ powder as the photocatalyst, much less bond fission occurred, since in-situ evolved hydrogen atoms transferred into substrates more easily on Pt nanoparticles than on TiO_2_ surface (as shown in Scheme 4). By preparing much smaller TiO_2_ nanoparticles with diameters ~50 Å, an apparent quantization effect appeared. The photohydrogenation efficiency of CH_3_C≡CH and HC≡CH was greatly enhanced with smaller TiO_2_ nanoparticles in comparison with bulk TiO_2_ particles [75]. The quantum yield was measured and the experimental result showed that as TiO_2_ nanoparticle became smaller, the quantum yield of the photohydrogenation of alkynes increased greatly. Yamataka et al. discovered that when hydrogen donor was changed from water vapor to alcohols such as ethanol or propan-2-ol, using platinized TiO_2_ as photocatalyst, much enhanced yield could be achieved for long-chain alkenes and alkynes transfer hydrogenation to alkanes [76]. This is a major advance for transfer hydrogenation by TiO_2_ photocatalysis. 

**Scheme 5:**
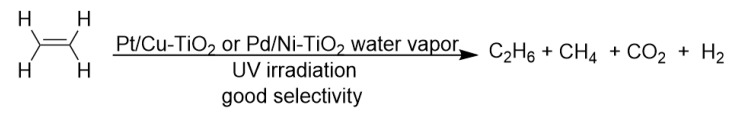
Bimetal nanoparticle-loaded-TiO_2_ photocatalyzed transfer hydrogenation of ethylene.

Apart from Pt-loaded TiO_2_, Baba et al. designed and synthesized the bimetal-deposited TiO_2_ and used it as an effective photocatalyst for transfer hydrogenation of ethylene with water vapor as HDC [77]. They discovered that a noble metal component such as Pt or Pd could act as hydrogen evolution and hydrogenation catalyst, while the second metallic component such as Ni or Cu acted as an efficient adsorbent for ethylene. Thus, bimetallic photocatalyst such as Pt/TiO_2_/Cu or Pd/TiO_2_/Ni was synthesized demonstrating higher selectivity for ethylene photohydrogenation to ethane other than C=C fission to CH_4_ and H_2_ (as shown in Scheme 5).

**Scheme 6:**
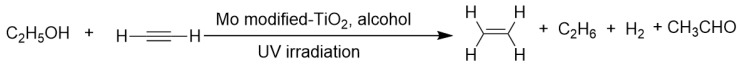
Molybdenum modified TiO_2_ photocatalyzed transfer hydrogenation of acetylene.

Kuntz et al. discovered that using poly-vinyl alcohol as a hydrogen donor, an electron-transfer reagent MoO_4_^2−^ acted as co-catalyst, acetylene transfer hydrogenation to ethane by TiO_2_ photocatalysis could be furnished (as shown in Scheme 6) [78]. They described that MoO_4_^2−^ ion adsorbing on TiO_2_ colloidal particle surface would promote the adjacent Ti(III) sites to execute four-electron reduction of acetylene through an electron-relay effect. Molybdenum played a similar role as noble-metal platinum to catalyze the hydrogen generation and hydrogenation. Later on, the same group further unearthed the mechanism behind the polynuclear Mo_2_ and Mo_3_ oxo species in assisting colloidal TiO_2_ nanoparticles to hydrogenate acetylene [79].

Both Mo_2_ and Mo_3_ oxo species promoted the photohydrogenation of acetylene to ethane by a 4-electron process on TiO_2_ colloidal particle surface. Besides, Mo_2_ oxo species also enhanced the ethylene to ethane 2-electron photoreduction process. In further studies, they designed a more efficient molybdenum-sulfur co-catalyst other than molybdenum-oxo for acetylene photohydrogenation by TiO_2_ colloidal particle [80]. MoS_4_^2−^ and the dimeric Mo_2_S_4_(C_2_H_4_S_2_)_2_^2−^ co-catalysts provided a higher turn-over number and quantum yield in comparison with its molybdenum-oxo analogues. In their ending work on this theme, Kuntz et al. designed and prepared the optimal molybdenum-based co-catalyst for acetylene photohydrogenation to ethane by TiO_2_ colloidal particle [81]. They combined the advantages of both Mo-oxo and Mo-sulfur co-catalysts and synthesized a dimeric co-catalyst Mo_2_O_x_S_x_(cys)_2_^2−^. A greatly improved quantum yields as high as 9.21% was achieved with the record high turn-over number (TON = 32.9) for acetylene photohydrogenation to ethylene by TiO_2_ catalyst system. The reason for this high catalytic activity was that the processes of electrons accepting from Ti (III) sites and electrons donating to the substrates bound to TiO_2_ surface were both greatly enhanced by this co-catalyst. This co-catalyst provided comparable catalytic possibility as platinum for this multi-electron transfer process of TiO_2_ photocatalyzed transfer hydrogenation.

**Scheme 7:**
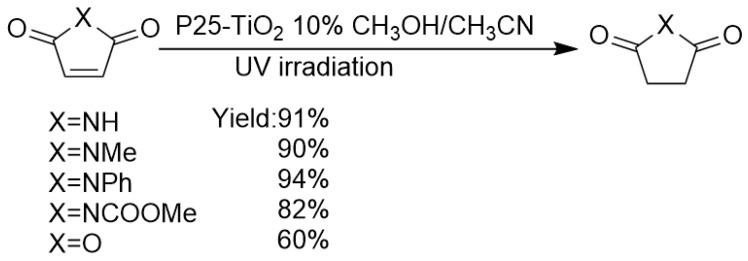
P25-TiO_2_ photocatalyzed transfer hydrogenation of maleimides and maleic anhydride using ethanol as a hydrogen source.

As an environmentally benign photocatalyst, TiO_2_ has little bio-toxicity and no secondary pollution. However, since TiO_2_ nanoparticle surface is extremely hydrated and possesses numerous polar Ti-OH groups, this material is extremely hydrophilic. Effectively adsorbing and converting non-polar and weak-polar functional group on TiO_2_ surface is very challenging. For hydrogenation of olefins with only C=C olefinic functional group, bare TiO_2_ photocatalyst without metal co-catalyst loading is generally considered to be futile. Although the benzene ring has weak adsorption interaction on polar TiO_2_ surface by the coordination of unoccupied Ti 3d orbital with benzene π-electron cloud, TiO_2_ photo-induced conduction-band electrons commonly could not photoreduce benzene C=C bond, because of its extreme inertness against the reductive transformation of the benzene ring. However, when C=C bond is conjugated to another polar functional group such as C=O bond, the polarity and redox potential of C=C bond is increased by a certain degree, because of the conjugation and induction effect of C=O bond. In this way, the 1,4-conjugate hydrogenations of C=C bond by TiO_2_ photocatalysis become possible if chemoselectivity can be successfully controlled. As shown in Scheme 7, Walton et al. reported that under UV light excitation, P25 TiO_2_ could catalyze the transformation of maleimides and maleic anhydride to succinimides and succinic anhydride using methanol as a hydrogen donor achieving good to excellent yields (yields ranging from 60% to 94%) [82].

Compared with earlier work by Kubokawa [72] and Anpo [74,75], the main development in this work was using alcohols instead of water vapor as hydrogen donors. This modification extremely enhanced the yield of hydrogenated products and chemoselectivity. Moreover, pristine maleimide bearing NH group can be selectively converted to succinimide with NH group intact. Furthermore, the authors expanded the substrate scope to N-aryl maleimide. Prolonged irradiation time was required and decreased isolated yield was obtained (9–79%). The key to the success of this transformation was the appropriate choice of a suitable class of olefin, i.e. maleimides, which has C=O group conjugated to C=C bond, leading to the non-polar olefinic bond’s diffusion, approaching and adsorption to TiO_2_ surface catalytic sites much easier, which greatly promoted the hydrogen transfer process. Moreover, suitable choice of hydrogen donor and solvent was also pivotal for the success of this reaction. Methanol hole-scavenger acted as the hydrogen donor, while the addition of acetonitrile decreased the nucleophilicity of methanol, thus reduced the possibility of the ring-opening side-reaction caused by methanol and methoxide species’ nucleophilic attack.

If C=C bond is not conjugated to C=O bond, its reduction becomes much more challenging due to the much-reduced redox potential [83,84]. Although pristine TiO_2_ photocatalyst could not initiate the reduction of styrene C=C bond, Pd-loaded TiO_2_ could realize this transformation achieving nearly quantitative yield [83]. In this case, Pd-H species played a very significant role to reduce C=C bond, since it possessed strong reducing power. However, the functional group tolerability of this method was not fully investigated.

Although TiO_2_ photocatalysis has accumulated some success in the transfer hydrogenation of C=C olefinic bond in maleimides, maleic anhydride and styrene, the reaction and substrate scopes are still very limited. The expansion of this method to the world of complex pharmaceutical and natural products synthesis still has long way to go. In the chemical markets, there are many desirable products used as pharmaceuticals, drugs and agrochemicals such as imatinib, bortezomib and imidacloprid, which are often required to be synthesized from the selective hydrogenation of unsaturated carbocyclic or heterocyclic structures of available precursors. Thus, developing more general methodology especially adapted to inactivated alkenes with an easily-reducible functional group is urgently needed. Moreover, to achieve selectivity including chemo-, regio- and enantioselectivity is also the future direction for transfer hydrogenations of C=C and C≡C bonds via TiO_2_ photocatalysis. To realize these goals, modification and crystal engineering of TiO_2_ nanomaterials, hybridizing TiO_2_ with other nanomaterials and the synergistic use of different catalysis modes would provide possible solutions.

## 3. Transfer Hydrogenation of C=O and C=N Bonds

Transfer hydrogenations of C=O bonds are extremely important since a variety of pharmaceuticals, agrochemicals and natural products are required to possess the bioactive asymmetric C-OH center, which can be conveniently prepared from the enantioselective reduction of the corresponding carbonyl compounds. Compared with transfer hydrogenations of C=C and C≡C bonds, TiO_2_ photocatalyzed transfer hydrogenations of C=O bonds have garnered more successes. The reason lies in that the hydrophilic TiO_2_ surface could effectively adsorb polar organic compounds by hydrogen bonds and coordination interaction of C=O lone-pair electrons with Ti 3d empty orbitals. In this way, polar C=O bonds could be facilely converted to C-OH bonds by interfacial electron/proton transfer, hydride transfer or hydrogen atom transfer delivered by HDC such as methanol, ethanol, i-propanol or triethylamine on TiO_2_ nanoparticle surface.

**Scheme 8:**
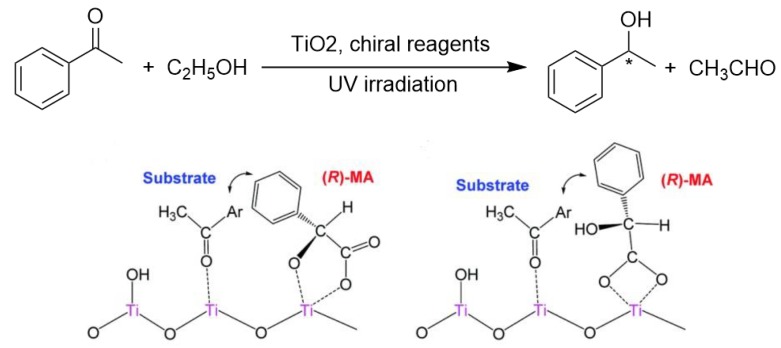
(**Top**): Chiral reagents modified-TiO_2_ photocatalyzed enantioselective transfer hydrogenation of acetophenone. (**Bottom**): Proposed models for (**left**) bidentate and (**right**) mono-dentate adsorption of (R)-mandelic acid (MA) and interaction between aromatic ketone and MA on the TiO_2_ surface.

Kohtani’s group has conducted a series of excellent work on TiO_2_ photocatalytic transfer hydrogenations of aryl carbonyl compounds using alcohols as hydrogen donors [55,85,86,87,88,89,90]. In their most recent work, even moderate enantioselectivity for aryl ketones’ conversion to chiral secondary aryl alcohols was realized (as shown in Scheme 8) [56]. Although previous work of König’s group had proved that the combination of TiO_2_ photocatalysis and imine organocatalysis could facilitate the asymmetric aliphatic aldehyde α-alkylation using α-bromomalonate as alkylating reagent [52], Kohtani’s discovery was the pioneering work in realizing heterogeneous asymmetric photocatalysis by fabricating a chiral TiO_2_ surface and directly deploying this surface for the asymmetric induction. In König’s work, TiO_2_ photocatalyst only acted as photo-redox catalyst without participating in chiral control [52]. However, Kohtani et al. accomplished the preparation of chiral TiO_2_ surface and applied this chiral surface for enantioselective control [56].

By adsorbing the chiral *R*-mandelic acid on TiO_2_ nanoparticle surface, the semiconductor surface became discriminative in transfer hydrogenation of acetophenone. This asymmetric transformation was furnished with 33% *ee* value. Although this *ee* value was not ideal, it demonstrated the possibility of fabricating asymmetric active sites on the TiO_2_ surface by adsorbing appropriate chiral molecules [52,56,91,92]. In this way, the asymmetric catalytic sites would impose the influence on transfer hydrogenations by providing sterically differentiated environment for ketone substrate to access these sites. Namely, the direction of attack from both photo-induced holes and electrons was of asymmetry.

**Scheme 9:**
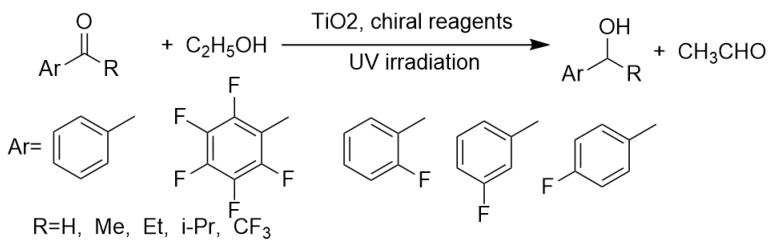
TiO_2_ photocatalyzed transfer hydrogenation of acetophenone derivatives.

In their earlier work, Kohtani et al. developed the transfer hydrogenation of aryl ketones and aldehydes to aryl secondary and primary alcohols. In 2010, this group initially reported the transfer hydrogenation of ketones by TiO_2_ photocatalysis using ethanol as a hydrogen donor (as shown in Scheme 9). Using ethanol or methanol as a hydrogen donor is very advantageous, since these short-chain molecules are volatile and easy to be separated from the mixture of the product by rotatory evaporation. 

Using commercial Degussa P25 TiO_2_ as photocatalyst under >340 nm irradiation, benzaldehyde and several acetophenone derivatives were transformed into the corresponding alcohols with good yields, respectively [85]. Aryl carbonyl compounds with less steric hindrance were hydrogenated preferentially. For instance, tert-butyl and iso-propyl phenyl ketones were reduced much more sluggishly and provided poorest yields (7% and 25%) in prolonged time compared with unsubstituted benzaldehyde and acetophenone. Moreover, this method could be extended to a variety of acetophenones with electron-donating and electron-withdrawing groups on phenyl ring resulting in good to excellent yield. Besides, bicyclic aryl ketones 2-acetonaphthalone could also be reduced with this method using either ethanol or i-propanol as HDC. However, this method could not be extended to aliphatic ketones. Cyclohexanone did not convert at all in this photocatalytic system, due to its much greater electron density and much lower redox potential. For diaryl ketone and aryl cyclic aliphatic ketone, this method was proved to be valid providing yields ranging from 78% to >99%. Later on, insightful mechanistic studies for this transformation were conducted. The different reduction modes of acetophenone and 2,2,2-trifluoroacetophenone were unveiled by systematic kinetic and adsorption studies [86,87]. Although 2,2,2-trifluoroacetophenone possessed higher redox potential, its reduction was slower than acetophenone. This phenomenon mainly originated from the ketone-hemiketal-ketal equilibrium. The rate-determining-step of the reduction was the hemiketal-ketone tautomerization. Only ketone form could be adsorbed on TiO_2_ surface by C=O lone-pair electrons with Ti 3d empty orbital. In 2,2,2-trifluoroacetophenone, the hemiketal and ketal are the major species, while for acetophenone, the ketone form is the major species. This subtle difference was uncovered and exploited to design more efficient transfer hydrogenation catalyst systems. 

**Scheme 10:**
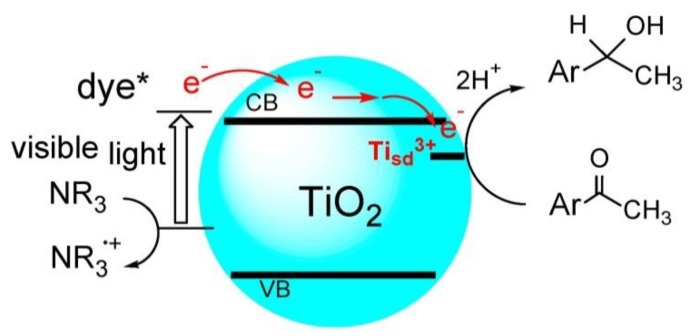
Dye-sensitized-TiO_2_ photocatalyzed transfer hydrogenation of acetophenones under visible-light irradiation.

Apart from UV-light excited transfer hydrogenation of ketones, visible-light could also be applied for hydrogenation of aryl ketones to secondary aryl alcohols. For example, Kohtani et al. reported that fluorescein and rhodamine B dye-sensitized TiO_2_ semiconductor photocatalyst could mediate the transfer hydrogenations of acetophenones and fluoro-substituted acetophenone derivatives under visible-light irradiation (as shown in Scheme 10) [55]. Initially, the dye molecules were anchored on the surface of TiO_2_ nanoparticles through carboxylate or phenolic linking groups. These adsorbed dye molecules were excited by the visible-light irradiation and the excited-state electrons on dye LUMO were injected into TiO_2_ conduction band. These electrons on TiO_2_ conduction band reduced acetophenones C=O group coupled with protons, while triethylamine substrates were oxidized by the ground state dye radical cations to continuously supply protons and electrons. Furthermore, the efficiency of this visible-light-sensitized protocol was greatly increased by the introduction of more absorptive dyes. A series of pristine and thiophene-modified coumarin derivatives were chosen as the photosensitizers for visible-light driven TiO_2_ photocatalytic transformation of acetophenone to 1-phenyl-ethanol [88]. The irradiation time to reach the same conversion was greatly shortened compared with the previous dye-sensitized system when appropriate hydrogen donor was chosen. Moreover, the author studied the kinetics of this transformation in detail [87]. Seven acetophenone derivatives were systematically investigated for this reductive transformation. Their redox potential, adsorptive energy on TiO_2_ surface and electron transfer efficiency were compared. From these experimental and theoretical results, a plausible mechanism was proposed. The Ti defect sites of TiO_2_ photocatalyst acted both as the adsorption and the electron transfer sites. The reaction rate and yield were mainly determined by the redox potential of acetophenones, as well as the adsorption interaction. Furthermore, the same group discovered that 2-fluoroacetophenone showed different product distribution in the TiO_2_ photocatalyzed transfer hydrogenation with fluoro-substituted acetophenones in spite of their similar structures [89]. 2-fluoroacetophenone provided defluorinated acetophenone, while 2,2,2-trifluoroacetophenone yielded 2,2,2-trifluoro-1-phenylethanol and 2,2-difluoroacetophenone formed both the defluorinated and the hydrogenated products. To explain this phenomenon, the plotting of the reaction rate versus the redox potential of different fluorinated acetophenones was conducted. The experimental results showed that the redox potential of fluorinated acetophenone determined the chemoselectivity of this transfer reduction. The chemoselective transfer hydrogenation of ketones and aldehydes to alcohols provided feasible functional group interconversion and the derivatization methods of pharmaceutically interesting molecules. 

**Scheme 11:**
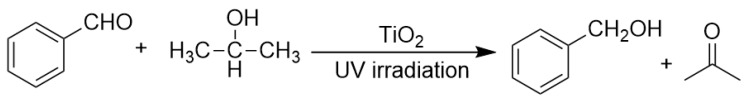
TiO_2_ photocatalyzed transfer hydrogenation of benzaldehyde.

Aldehydes could also be directly transformed to primary alcohols by TiO_2_ photocatalyzed transfer hydrogenation [93]. Li et al. reported that in the presence of HDC such as ethanol or i-propanol, both aromatic aldehydes and aliphatic aldehydes could be reduced to the corresponding primary alcohols in a TiO_2_ suspending solution under near-UV irradiation (as shown in Scheme 11) [93]. By the comparison of the isotope tracing experiments results and kinetics curve analysis of different functionalized benzaldehyde derivatives, they demonstrated that this reaction proceeded through a stepwise SET (single electron transfer) mechanism accompanied with simultaneous protonation other than direct hydrogen atom transfer pathway. Besides alcohols, oxidable amines are also excellent hole-scavenger and hydrogen source for some dye-modified TiO_2_ photocatalytic system with lower redox potential and oxidability, since amines are usually easier to donate electron than alcohols. 

**Scheme 12:**
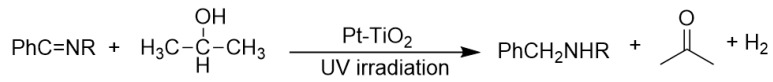
TiO_2_ photocatalyzed transfer hydrogenation of Schiff bases.

Not only carbonyl C=O could be effectively reduced to the corresponding C-OH bonds, but imine C=N bonds could also be photohydrogenated by TiO_2_ photocatalyst. Ohtani et al. reported that under UV irradiation in a Pt-TiO_2_ suspending solution, *N*-benzylidenebenzylamine and *N*-benzylideneaniline could be hydrogenated to the corresponding secondary amine dibenzylamine and *N*-benzylaniline with the simultaneous photo-oxidation of 2-propanol by TiO_2_ photo-induced hole, respectively, in high selectivity and yield (as shown in Scheme 12) [60].

This transfer photo-hydrogenation occurred in a stepwise SET followed by protonation pathway the same as the previous Li’s example for benzaldehydes. They discussed the influence of Pt-loading, the addition of desiccating reagents, acid and the preparation condition of the photocatalysts to the overall selectivity and yield. An earlier report by Kagiya et al. demonstrated that both the symmetrical, unsymmetrical secondary and the tertiary amine could be synthesized by the Pt-TiO_2_ photocatalyzed transfer hydrogenations of in-situ formed imine intermediate which were generated from the condensation of primary amines and alcohols [94,95,96]. Apart from aldehyde and dihydrogen, no other by-products were formed. With the introduction of an alcoholic solvent, unsymmetrical secondary and tertiary amines were synthesized from Pt-TiO_2_ photocatalyzed hydrogenation of imines. In this case, imines were generated from the condensation of primary amines and aldehydes, which were formed through oxidation of the alcohol solvent by photo-induced holes [94]. Moreover, Ohtani et al. reported that α,ω-diamino carboxylic acids could be transformed to five- or six-membered cyclic imino acids via Pt-TiO_2_ photocatalysis [95]. They described that α,ω-diamino carboxylic acids were initially oxidized by photo-induced holes to the corresponding α-keto acid or ω-aldehyde intermediates bearing carbonyl and amino groups. Condensation between carbonyl and amino groups generated the cyclic imine intermediate. The following reduction by conduction band electrons in Pt-TiO_2_ along with protonation provided the final cyclic imino acid products. Pt-loaded TiO_2_ particles played two pivotal roles in these C=N transfer hydrogenations. The first was to act as proton reduction site to generate dihydrogen. The second was the hydrogenation site to help the dihydrogen molecule reduce C=N bond to amino compounds. 

**Scheme 13:**
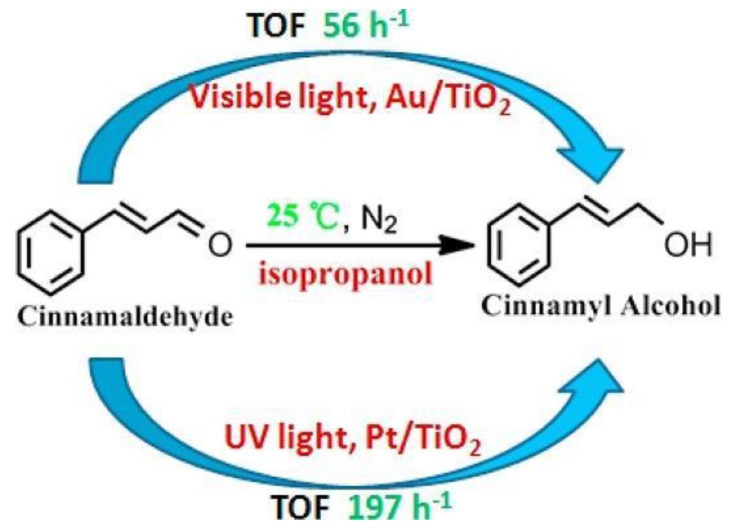
Au/Pt-TiO_2_ photocatalyzed transfer hydrogenation of cinnamaldehyde to cinnamyl alcohol.

When polar C=O was conjugated to non-polar C=C bond, Au/TiO_2_ or Pt/TiO_2_ could act as chemoselective photocatalyst to reduce C=O bond to C-OH with C=C bond intact. Li et al. reported that under 365 nm UV irradiation, Pt/TiO_2_ photocatalyst provided excellent performance with record high apparent quantum yield (AQE) for photocatalytically selective reduction of cinnamaldehyde to cinnamyl alcohol (as shown in Scheme 13) [57]. Besides, Au/TiO_2_ also achieved satisfactory selectivity at high conversion under visible-light irradiation (> 420 nm).

They postulated a six-membered transition-state model to illustrate this chemoselectivity. Under UV or visible-light irradiation, TiO_2_ photo-induced holes oxidize the electron and hydrogen donor i-propanol yielding active hydrogen species on metal particles. The C=O group adsorbed on TiO_2_ surface and occupied the vicinal position to the metallic nanoparticle which adsorbed hydrogen. This vicinity facilitates the hydrogen transfer from metal to C=O group on TiO_2_ surface. Once alcohol is adsorbed on the TiO_2_ surface by hydrogen bonds, the photo-induced holes migrate to TiO_2_ surface and then oxidize these adsorbed alcohol molecules to generate protons and aldehyde product. The protons combine with TiO_2_ photo-induced electron on Au and Pt anchoring sites yielding hydrogen atoms or dihydrogen. These active hydrogen species generated by Au or Pt nanoparticle will reduce C=O bonds. Moreover, this article also indicated that alcohol oxidation and the simultaneous carbonyl reduction both occurred at the Au- or Pt-adsorbed TiO_2_ site.

Apart from plasmonic metal-loaded TiO_2_ nanoparticles photocatalyst systems for chemoselective C=O reduction over thermodynamically more favorable C=C reduction [87], organic molecules-modified TiO_2_ nanoparticles could also solve this chemoselectivity issue. Dihydroxynaphthalene chelated TiO_2_ nanoparticles behaved as efficient photocatalyst for visible-light induced transfer hydrogenation reduction of benzaldehydes to the corresponding benzyl alcohols with the maintaining of easily-reducible functional group kept intact [97]. By a simple impregnation procedure, the as-synthesized dihyroxynaphthalene-modified TiO_2_ nanoparticles promoted the transfer hydrogenation of a variety of benzaldehydes with chloro, bromo, iodo, acetyl and cyano groups kept intact [97]. This high selectivity originated from attenuated reducing power of dye-sensitized conduction-band electrons compared with direct UV-light induced conduction-band electron. 

Using TiO_2_ and modified TiO_2_ nanomaterials, a series of compounds with C=O and C=N bonds could be photohydrogenated to the corresponding saturated alcohols and amines products, respectively. In this way, TiO_2_ photocatalysis provides one of the most important means for reductive transformation of polar unsaturated compounds to the corresponding saturated ones using short-chain alcohols or amines as hydrogen sources. This method could be applied in the production of alcohols and amines molecules, which are often very important products, key intermediates and candidates for drugs, agrochemicals, flavors and fragrances. The most economically interesting products such as chiral drugs with saturated heterocyclic structures could be synthesized by this prospective methodology. TiO_2_ photocatalyzed transfer hydrogenation strategies to reduce C=O and C=N bonds with other sensitive and bio-active groups kept intact has the potential to enlarge the synthetic toolbox of building complex molecule, shorten synthesis steps and maximize economic benefit. 

## 4. Transfer Hydrogenation of N=O Bonds

**Scheme 14:**
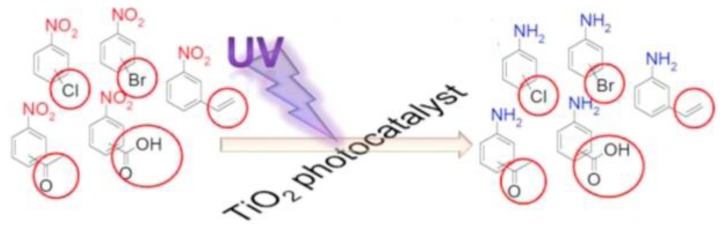

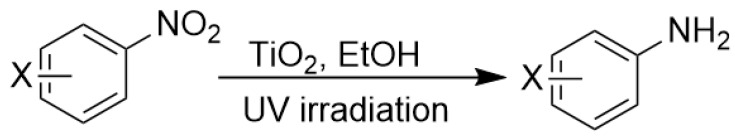
TiO_2_ photocatalyzed transfer hydrogenation of nitrobenzenes using alcohol as a hydrogen source.

The amino group plays pivotal role in many important drug molecules and agrochemicals [98]. The most convenient method to prepare the amino groups is the selective reduction of nitro groups. However, this transformation is challenging due to the difficulty in controlling selectivity [99]. Common reductant such as NaBH_4_ or LiAlH_4_ often leads to the mixture of nitroso, amino, azo, azoxy and hydrazine products which are often difficult to separate requiring time-consuming column chromatographic operations. Generally, thermocatalytic transformations of nitro to the amino group often require noble metal catalysts Pd and explosive dangerous gaseous H_2_ [100]. To meet the demand and principle of green chemistry, TiO_2_ semiconductor photocatalysis provided a very promising means of nitro to amino compounds based on transfer hydrogenation mechanism (as shown in Scheme 14) [101].

As early as 1993, Li et al. discovered that when TiO_2_ nanoparticles suspended in an ethanol solution, nitrobenzenes could be transformed into the corresponding amino compounds in good isolated yields under UV irradiation [102]. The high efficiency and selectivity was thermodynamically controlled by the difference of reduction potential between nitro compounds and TiO_2_ conduction band electrons. The conduction band potential of TiO_2_ (−0.85 V versus SCE) in acetonitrile, is more negative than p–nitroacetophenone (–0.1 V versus SCE).

Brezová et al. systematically studied the solvent influences on the efficiency of TiO_2_ photocatalyzed chemoselective 4-nitrophenol reduction to 4-aminophenol [61]. They discovered that viscosity, polarity, polarizability and polarity/polarizability ratio all influence the efficiency. A solvent with the minimum viscosity, the maximum polarity, polarizability and the highest polarity/polarizability ratio, namely, methanol provided the highest reaction rates when other conditions were identical. 

Ferry et al. conducted the studies to confirm the real reducing species of TiO_2_ photocatalyzed aromatic nitro and aliphatic nitro compounds reduction: Alkylhydroxy radicals or TiO_2_ conduction band electrons [63,103]. From the carbon and nitrogen mass-balance experiments, they discovered that hydroxylamine may possibly be the pivotal intermediates. By comparing the kinetics curve of nitro compounds with different substitution groups, the authors deduced a counterintuitive point that nitro compounds with electron-withdrawing group would retard the transformation of nitro to the amino group. Moreover, from the kinetics plot of the reaction rate of nitro compounds versus the redox potential of different alcohols such as methanol and i-propanol, TiO_2_ conduction band or trapped-state electrons other than α-alkylhydroxy should be known as responsible for reductive transformation, albeit different nitro compounds showed almost the same kinetics constant in the presence of either methanol or i-propanol. 

**Scheme 15:**
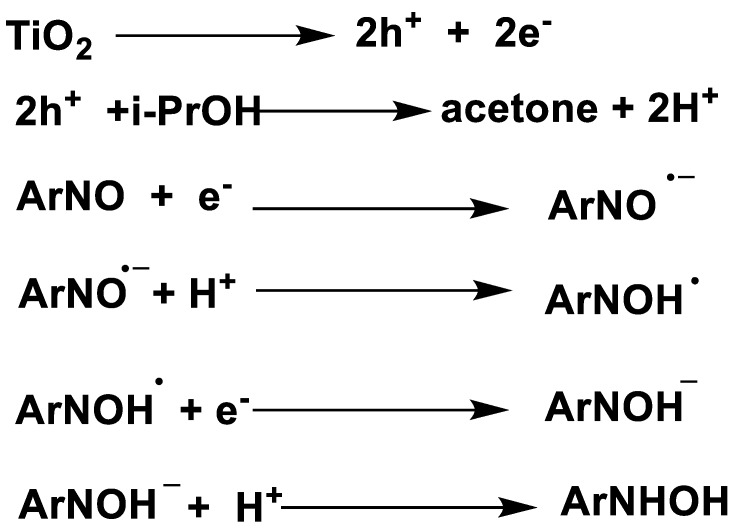
Mechanism of aromatic nitroso compounds transfer reduction by TiO_2_ photo-generated electron and proton in i-PrOH as demonstrated by the EPR studies.

Using electron-paramagnetic-resonance (EPR) techniques as the characterization tool, Brezová et al. [62] studied a number of key intermediates in the TiO_2_ photocatalytic transfer hydrogenation of nitrosobenzene derivatives. Taking nitrosobenzene as an example, they demonstrated the mechanism of TiO_2_ photocatalytic transfer hydrogenation of aromatic nitroso compounds by i-propanol as HDC, in which the radical species were characterized during the photocatalysis process. (as shown in Scheme 15). Initially, photo-induced electrons on TiO_2_ conductor band reduce nitrosobenzene to form mono-valence radical anion along a SET route. After protonation, N-OH• radical was generated. During the EPR measurements, no spin-trapping adducts of alkoxy, hydroxylalkyl and alkyl free-radicals were observed. Moreover, deuterium labelling experiments using CD_3_OD/toluene mixed solvent with other conditions identical were conducted. In agreement with the authors’ proposition, N-OH• was characterized by its different hyperfine splitting in comparison with N-OH•.

Furthermore, the authors studied other nitrosobenzene derivatives (2-nitrosobenzene, nitrosodurene, 2,3,4,5-tetramethylnitrosobenzene, 2,4,6-tritert-butylnitrosobenzene, 3,5-dibromo-4-nitrosobenzenesulfonate and 2-methyl-2-nitrosopropane) photo-induced transfer hydrogenation reductions with alcohol (methanol, ethanol and i-propanol) in TiO_2_ slurry by EPR technique. All the results obtained the similar conclusion of transfer hydrogenation, that is, photo-induced hole oxidation of alcohols produces protons and corresponding aldehydes products in terms of 2e-ET, while the photo-induced electrons reduced nitrosobenzenes along a stepwise SET route.

Makarova et al. prepared surface modified TiO_2_ nanomaterials with L-arginine, lauryl sulfate and salicylic acid as modifier [104,105,106]. After surface modification with arginine, nitrobenzene adsorption on TiO_2_ surface was enhanced and the transformation of nitrobenzene to aniline was greatly accelerated by this modification strategy. From 10 K, 120 K and 200 K varied-temperature EPR experiments, the authors uncovered that upon surface arginine modification, the surface trapped electron signals were absent in the EPR spectra, which differed greatly with bare TiO_2_ and other two modified samples. This phenomenon was mainly reasoned to derive from the fairly good electron coupling between TiO_2_ and arginine through the anchoring group of ammonium and carboxylate group of L-arginine with TiO_2_ surface oxygen lone-pair electron of Ti-O and Ti 3d unoccupied orbital. This electron coupling interaction facilitated TiO_2_ trapped electrons transfer to nitrobenzene more efficiently, leading to the highest yield for arginine modified TiO_2_. Tada et al. loaded Ag clusters onto TiO_2_ nanoparticles surface to realize a reasonable delivery photocatalytic reaction system (RDPRS) for the transformation of nitrobenzene to aniline [107]. They discovered that upon Ag cluster loading, the activity and the aniline product selectivity were both drastically increased. From the adsorption experiments investigation, the authors discovered that nitrobenzene was selectively adsorbed on Ag cluster rather than on bare TiO_2_ and the aniline product was neither adsorbed by Ag nor by TiO_2_. This difference in adsorptivity facilitated the desorption of aniline from TiO_2_ surface against further unselective over-oxidation. Also, the surface plasmonic effect of Ag particles enhanced the electron transfer from TiO_2_ conduction band to the nitrobenzene compounds, which accelerated the hole oxidation by means of the rapid transfer of conduction band electron. 

Zhang et al. systematically investigated different factors influencing the yield and selectivity of *p*-chloronitrobenzene reduction to p-chloroaniline [108]. They discovered that solvent played pivotal role in increasing product yield of *p*-chloroaniline. HCOOH/i-propanol as a hydrogen donor and solvent provided the best results achieving quantitative yield (99.2%). i-propanol is the best solvent and hydrogen donor, since i-propanol has the largest steric hindrance and highest redox potential among small molecule alcohols. This means that the TiO_2_ photo-induced hole oxidation of i-propanol is more difficult and slower than other hydrogen donor, which possibly provided a milder reduction condition and thus received good selectivity and yield of the transfer hydrogenation. 

Kominami et al. studied the TiO_2_ photocatalytic reduction of nitrobenzene using oxalic acid as green transfer hydrogenation reagent in aqueous solution [109]. They discovered that in the presence of a small partial pressure O_2_ (5%), higher yield of aniline was achieved than in the absence of O_2_. Although the authors did not provide the essential role of this small amount of O_2_, we proposed that the low partial pressure O_2_ possibly acted as an electron shuttle or energy transfer reagent through ROS species that facilitated the electron and energy transfer processes during the total photocatalytic transfer hydrogenation. With the optimal conditions in hand, an excellent 95% yield of aniline was realized using oxalic acid hydrogen source in the aqueous solution. The only by-product was the oxidation product of oxalic acid-CO_2_. 

**Scheme 16:**
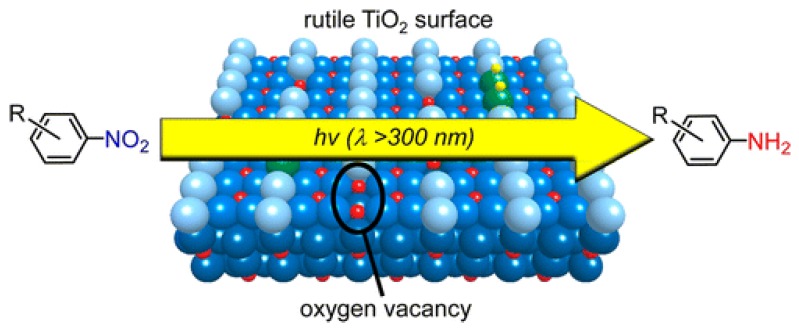
Rutile-TiO_2_ photocatalyzed nitrobenzenes transfer hydrogenation to anilines.

Shiraishi et al. utilized rutile TiO_2_ nanoparticles as a photocatalyst for effective nitrobenzene reduction. (as shown in Scheme 16) [110]. For rutile TiO_2_, more Ti^3+^ species on the surface of TiO_2_ were generated upon UV irradiation than anatase. The Ti^3+^ species played a dual role both as the nitrobenzene adsorption and the electron trapping sites. In the presence of alcohol as a hydrogen source, a variety of nitrobenzene derivatives could be transformed into the corresponding anilines in excellent yield (>94%) and quantum yield (25%) with another reducible functional group intact (as shown in Table 1). 

Not only UV-light could induce the TiO_2_ photocatalytic transfer hydrogenation of nitrobenzenes to anilines, but also lower energy visible green light could excite dye-sensitized TiO_2_ to catalyze this transformation. König et al. prepared a TiO_2_/Ru-N_3_ metal-oxide semiconductor/transition metal ion/dye ternary composite catalyst system [58]. 

Under green-light or sunlight irradiation, excited state N_3_ dye transferred its electrons to TiO_2_ conduction band, these in-situ generated TiO_2_ conduction band electrons reduced the vicinal Pt, Pd or Au ion to the corresponding metal colloidal particles. These metal particles behaved as reaction sites for hydrogen generation and hydrogenation of nitrobenzenes. Triethanolamine acted as the final electron/hydrogen donor to regenerate the ground state N_3_ dye. This visible-light-induced transformation demonstrated good substrate tolerability with many functional groups on nitrobenzene rings kept intact under the standard photoreduction conditions. Moreover, this method provided an excellent yield for anilines.

Using TiO_2_ photocatalytic transfer hydrogenation for the transformation of nitro to amino compounds has already gained great advances since the beginning of the 1990s. A number of nitrobenzene derivatives could be transformed into the corresponding anilines under UV, sunlight or even visible-light irradiation under pristine TiO_2_, meta-loaded TiO_2_ and dye-sensitized TiO_2_ nanomaterials. EPR and surface-sensitive spectrometry such as attenuated total reflection infrared (ATR-FTIR) and diffuse reflectance infrared Fourier transform spectroscopy (DRITS-FTIR) provided much information on the reaction pathways of this heterogeneous photocatalytic reaction [62,63,103,104]. However, there are yet some challenging issues in this field to be addressed. For instance, how to enhance the optimal yield, utilize the total solar spectrum to near-infrared region and meliorate the functional group tolerability and extend the application to more complex molecules are interested in medicinal chemistry and natural product chemistry. All of these called for a more insightful understanding of the reaction mechanism.

## 5. Conclusions

We have conducted a review of paragon examples of TiO_2_ photocatalyzed transfer hydrogenations. Although still in its budding period in comparison with its currently prevalent applications in water-splitting, dye-sensitized-solar-cell, aqua system and air atmosphere pollutant decomposition [111], TiO_2_ photocatalysis has already exhibited the potential in organic synthesis [112], especially in transfer hydrogenation based on safe and cheap HDC (hydrogen donor compounds). Various unsaturated bonds, including C=C, C≡C, C=O, C=N, N=O bond, could be transformed into the corresponding saturated C-C, C-O, C-N and N-H bonds, respectively. Compounds with non-polar olefin and acetylene, polar aldehyde, ketone, imine and nitro functional groups can be smoothly reduced by TiO_2_ photocatalysis using transfer hydrogenating reagents, such as water, alcohols, and amines. By means of this strategy, these unsaturated bonds in functionalized organic substrates could be transformed into the useful saturated moieties in diverse complex organic functional molecules. This research topic has already become a hot and active area being focused by both photocatalytic and synthetic field. Comparing with homogeneous Ru-, Ir-polypyridyl complex and organic dye photocatalysts, however, TiO_2_ photocatalysis demonstrates a fairly narrow substrate scope and limited reaction types and still has much space to improve. Current transfer hydrogenations by TiO_2_ photocatalysis still lack selectivity, especially in enantioselectivity. The latter is the widest gap between the state-of-the-art TiO_2_ photocatalyzed transfer hydrogenation and the future requirement for this methodology. Although there are sparse reports of chiral TiO_2_ surface photocatalyzed transfer hydrogenation of acetophenone, the *ee* value is still very low. The scope of TiO_2_ photocatalyzed asymmetric transfer hydrogenation needs to be widened. Only by the continuous efforts in the optimization of reaction conditions and materials engineering of photocatalysts could this asymmetric photocatalyzed transfer hydrogenation be applied in laboratorial and industrial scales enantiomer’s synthesis. By more elaborate designing of TiO_2_ nanomaterials itself or artful choice of co-catalysts, additives, and reaction parameters, this research area will provide us with greener, safer, more environmentally benign, efficient designs and strategies to supplement the traditional transition-metal complexes catalysts and organocatalysts for transfer hydrogenation of unsaturated compounds.

## Figures and Tables

**Table 1:** Rutile-TiO_2_ photocatalytic transfer hydrogenation of nitrobenzenes with i-propanol.

Entry	Substrate	Solvent	t/h	Conversion/%	Product	Yield/%
1	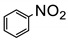	i-PrOH	6	>99	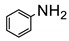	>99
2	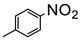	i-PrOH/toluene (1/9)	4	>99	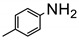	98
3	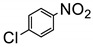	i-PrOH/toluene (9/1)	5	>99	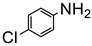	94
4	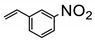	i-PrOH/toluene (5/5)	7	>99	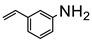	98
5	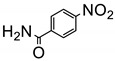	i-PrOH/toluene (1/9)	6	>99	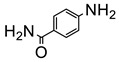	94
6	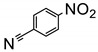	i-PrOH/toluene (1/9)	6	>99	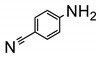	97
7	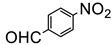	i-PrOH/toluene (1/9)	6	>99	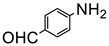	94

Reaction conditions: Substrate (50 μmol), rutile-TiO_2_ catalyst (10 mg), solvent (5 mL), temperature (303 K), N_2_ (1 atm), Xe lamp (λ > 300 nm).
